# The Toll–Like Receptor 2/6 Agonist, FSL–1 Lipopeptide, Therapeutically Mitigates Acute Radiation Syndrome

**DOI:** 10.1038/s41598-017-17729-9

**Published:** 2017-12-11

**Authors:** Cathryn J. Kurkjian, Hao Guo, Nathan D. Montgomery, Ning Cheng, Hong Yuan, Joseph R. Merrill, Gregory D. Sempowski, W. June Brickey, Jenny P.-Y. Ting

**Affiliations:** 1 Lineberger Comprehensive Cancer Center, University of North Carolina, Chapel Hill, NC USA; 2 Department of Pathology and Laboratory Medicine, University of North Carolina, Chapel Hill, NC USA; 3 Oral Biology Curriculum, School of Dentistry, University of North Carolina, Chapel Hill, NC USA; 4 Department of Radiology, University of North Carolina at Chapel Hill, Chapel Hill, NC USA; 5 Biomedical Imaging Research Center, University of North Carolina at Chapel Hill, Chapel Hill, NC USA; 6 Duke Human Vaccine Institute, Duke University, Durham, NC USA; 7 Department of Genetics, University of North Carolina, Chapel Hill, NC USA

## Abstract

Risks of radiation exposure from nuclear incidents and cancer radiotherapy are undeniable realities. These dangers urgently compel the development of agents for ameliorating radiation–induced injuries. Biologic pathways mediated by myeloid differentiation primary response gene 88 (MyD88), the common adaptor for toll–like receptor (TLR) and Interleukin–1 receptor signaling, are critical for radioprotection. Treating with agonists prior to radiation enhances survival by activating TLR signaling, whereas radiomitigating TLR–activating therapeutics given after exposure are less defined. We examine the radiomitigation capability of TLR agonists and identify one that is superior for its efficacy and reduced toxic consequences compared to other tested agonists. We demonstrate that the synthetic TLR2/6 ligand Fibroblast–stimulating lipopeptide (FSL–1) substantially prolongs survival in both male and female mice when administered 24 hours after radiation and shows MyD88–dependent function. FSL–1 treatment results in accelerated hematopoiesis in bone marrow, spleen and periphery, and augments systemic levels of hematopoiesis–stimulating factors. The ability of FSL–1 to stimulate hematopoiesis is critical, as hematopoietic dysfunction results from a range of ionizing radiation doses. The efficacy of a single FSL–1 dose for alleviating radiation injury while protecting against adverse effects reveals a viable radiation countermeasures agent.

## Introduction

Deliberate or accidental radiation release in the cases of terrorism and nuclear warfare or energy plant and waste facility explosions respectively, will expose a diverse population to various degrees of penetrating ionizing radiation. Therefore, medical interventions that can be administered to counteract injury associated with radiation are critically needed^1,2^. As directed by the U.S. Department of Homeland Security (https://www.dhs.gov/topic/nuclear-security)^3^ and recommended by an NIH panel (https://www.niaid.nih.gov/topics/radnuc/program/Pages/FocusedResearchDevelopment.aspx)^4^, immediate goals for radiation countermeasures include the development and expansion of products that effectively prevent or treat radiation injury^5^. An ideal medical intervention following radiation is defined by the following properties: (1) administration at 24 hours or more post exposure, (2) independent of sex or age, and (3) application to individuals exposed to a variety of radiation doses.

Acute radiation syndrome (ARS) is a disease state that occurs following partial or whole body exposure to ionizing radiation. ARS can be further characterized into hematopoietic (H–ARS), gastrointestinal (GI) and cerebrovascular syndromes, which develop based on the type, dose and rate of radiation received. H-ARS is observed at lower doses of radiation (200–600 rad), but also persists in tandem with GI and cerebrovascular syndromes, which occur only upon exposure to higher doses (600–1000 rad). Medical treatments, therefore, targeting hematopoiesis would be vital during a mass casualty event.

Replenishment of hematopoietic sites is critical for recovery following radiation exposure. Regeneration of the hematopoietic system occurs successfully through multiple mechanisms^6^. Hematopoiesis is driven, in part, by various growth factors, including granulocyte colony-stimulating factor (G-CSF), erythropoietin (EPO) and thrombopoietin (TPO). These factors are thought to drive proliferation of granulocytes, erythroid cells, and megakaryocytes, respectively, though some degree of cross-regulation may occur between lineages. Beyond driving granulocyte proliferation, G-CSF can also impact the function of lymphoid lineages^7^. In addition to growth factors, TLR receptors found on progenitor cells utilize MyD88-dependent mechanisms to drive cellular repopulation following insults to the hematopoietic system^8^. Furthermore, regulation of hematopoiesis can be driven by upregulating growth factors through TLR signaling^9,10^. Importantly, a correlation between G-CSF and TLR2–dependent signaling has been demonstrated^10,11^.

MyD88–dependent TLR signaling is immune stimulatory and has been shown to induce protective mechanisms against radiation^12^. Since the seminal articles reported the unexpected finding that the TLR5 agonist flagellin provides radioprotection^13,14^, other TLR ligands have been tested as radioprotectors through administration prior to partial or total body irradiation. In most cases, these studies demonstrated improved survival and protection, with accelerated hematopoiesis and/or inhibition of apoptosis within the GI tract^13,15–19^. Such radioprotection has been observed using ligands specific to TLR2, TLR3, TLR4 and TLR5^1,2,13,15–25^. However, the majority of these studies examined the use of ligands prior to radiation exposure, characterizing radioprotective mechanisms, whereas activity of these TLR ligands as radiomitigators given post radiation exposure is less understood.

The study of TLR2 ligands has enhanced the development of novel radiation countermeasures through broadening the understanding of how these immune stimulatory agonists function^16,19,21,26^. Indeed, the use of lipopeptide–based TLR2–mediated ligands is thought to be critical for driving cytokine– and chemokine–based responses that target H–ARS^19,21,22^. The TLR2/6 ligand, Fibroblast–stimulating lipopeptide FSL–1 (Pam2CGDPKHPKSF), contains a diacylglycerol structure similar to Pam2CSK4 and has been shown to play critical roles in immune cell maturation, Th2 immunity and protection from infections^27–30^. Whether FSL–1 serves a role in counteracting radiation–induced injury has not been determined.

This study focuses on elucidating the radiomitigation effects of FSL–1, a different class of TLR ligand that activates TLR2/6, and understanding its role in improving hematopoietic responses associated with ARS, thereby demonstrating capacity to function as medical countermeasures against radiation. Herein, we examine the ability of a variety of TLR ligands to confer protection from H–ARS when administered at least 24 hours post radiation exposure. We identify the TLR2/6 ligand FSL–1 as a potent radiomitigator that was superior to all others we tested, demonstrating exceptional efficacy and diminished adverse effects. We present data showing that the radiomitigation activity of FSL–1 is sex–independent and MyD88–dependent. Furthermore, we determine that a single administration of FSL–1 positively impacts hematopoiesis and induces G–CSF production following acute radiation injury. Overall, these data reveal that the immune stimulatory effects of FSL–1 mitigate radiation injury by accelerating hematopoietic recovery at multiple sites following radiation exposure and promoting survival. Lastly, this report illuminates the benefits of FSL–1 as a potential therapeutic for victims of radiation exposure by nuclear incidents as well as for patients receiving radiotherapy.

## Results

### The radiomitigation capabilities of TLR ligands are tested

To examine the role of TLR ligands as radiomitigators, C57BL/6 mice were exposed to ionizing gamma radiation (9.2 Gy) from a cesium source, followed by administration of TLR ligands via intraperitoneal (i.p.) injections one day after radiation. We examined the impact of CpG–ODN2395 (CpG, TLR9 agonist), Flagellin FliC (Fla, TLR5 agonist), MPLA (TLR4 agonist) or FSL–1 (Pam2CGDPKHPKSF, TLR2/6 agonist) in comparison to physiological water (no treatment, NT). Dosing of each ligand was chosen based on previous work^14,17,18,21,22,31^ and conducting experiments with a reasonable number of experimental subjects.

**Figure 1:**
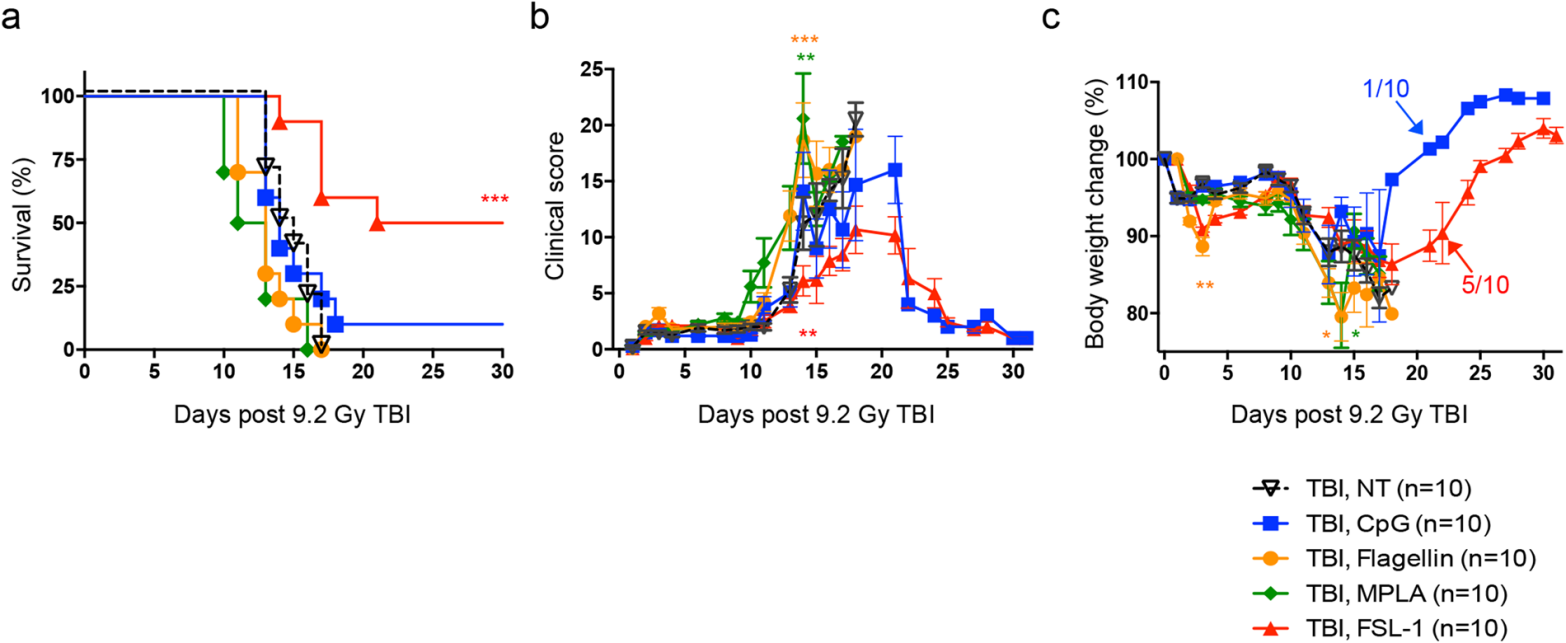
Treatment with TLR ligands impacts survival of irradiated mice. Male C57BL/6 mice were irradiated (9.2 Gy TBI) and 24 hours later, received a single i.p. injection of physiological water (no treatment, NT), 2.5 mg/kg CpG–ODN2395 (CpG), 0.2 mg/kg Flagellin (Fla), 0.25 mg/kg MPLA, or 0.25 mg/kg FSL–1 (*n* = 10 per group). (**a**) Survival over 30 days post TBI, (**b**) clinical score and (**c**) percent body weight change of each treatment group was compared to irradiated, non–treated mice (TBI, NT). Data are representative of 2 independent experiments and are depicted as mean ± s.e.m. *P < 0.05, **P < 0.01 or ***P < 0.001 between treated and TBI, NT mice, with colors correlating to treatment group. Arrows indicate time post TBI in which remaining mice in group began to recover.

Thirty–day survival of non–treated male C57BL/6 mice exposed to 9.2 Gy total body irradiation (TBI) varied between replicate experiments from 0–50% (mean 22.4%; Table 1). Control mice succumbed to the effects of radiation by approximately 2 weeks post TBI (Fig. 1a), as defined by weight loss greater than 25% and/or a clinical score greater than 15 that encompasses seven body parameters (shown in Supplementary Table S1). Only 1 of 10 mice treated with CpG (10%) survived for 30 days post TBI compared to 0% of non–treated mice (Fig. 1a). The clinical score and weight change of CpG–treated mice was similar to that of control mice (Fig. 1b,c). Fla– or MPLA–treated mice did not survive beyond 17 days after TBI. Mortality of Fla– or MPLA–treated mice was associated with an increased clinical score and substantial weight loss (Fig. 1b,c).

**Table 1:** Survival of non–treated or FSL–1–treated irradiated mice.

Expt.	*n*	No treatment		FSL−1 (0.25 mg/kg)		
		No. Surviving for 30 days	Survival (%)	*n*	No. Surviving for 30 days	Survival (%)
1	20	6	30.0	20	19	95.0
2	4	2	50.0	6	6	100.0
3	5	0	0.0	5	5	100.0
4	10	0	0.0	10	5	50.0
5	5	0	0.0	8	5	62.5
6	7	2	28.6	5	2	40.0
7	10	4	40.0	9	8	88.9
8	6	1	16.7	3	3	100.0
*Total*	*67*	*15*	22.4*	*66*	*53*	80.3*

*P < 0.0001 between No treatment and FSL−1 treatment for overall mean survival (%).

By contrast to the high mortality of mice treated with other TLR agonists, 30 day survival of FSL–1–treated mice ranged from 50–100% (mean 80.3%) over a course of 8 independent experiments (Table 1). In experiments directly comparing various TLR ligands, FSL–1–treated mice showed superior survival relative to mice treated with other agonists (Fig. 1a) (P = 0.0005). Increased survival was associated with notably decreased clinical score compared to non–treated (NT) irradiated mice (Fig. 1b), although no difference in weight loss was observed during the first 2 weeks after radiation (Fig. 1c).

### Radiomitigation by FSL–1 results in fewer adverse effects than other TLR2 ligands

**Figure 2:**
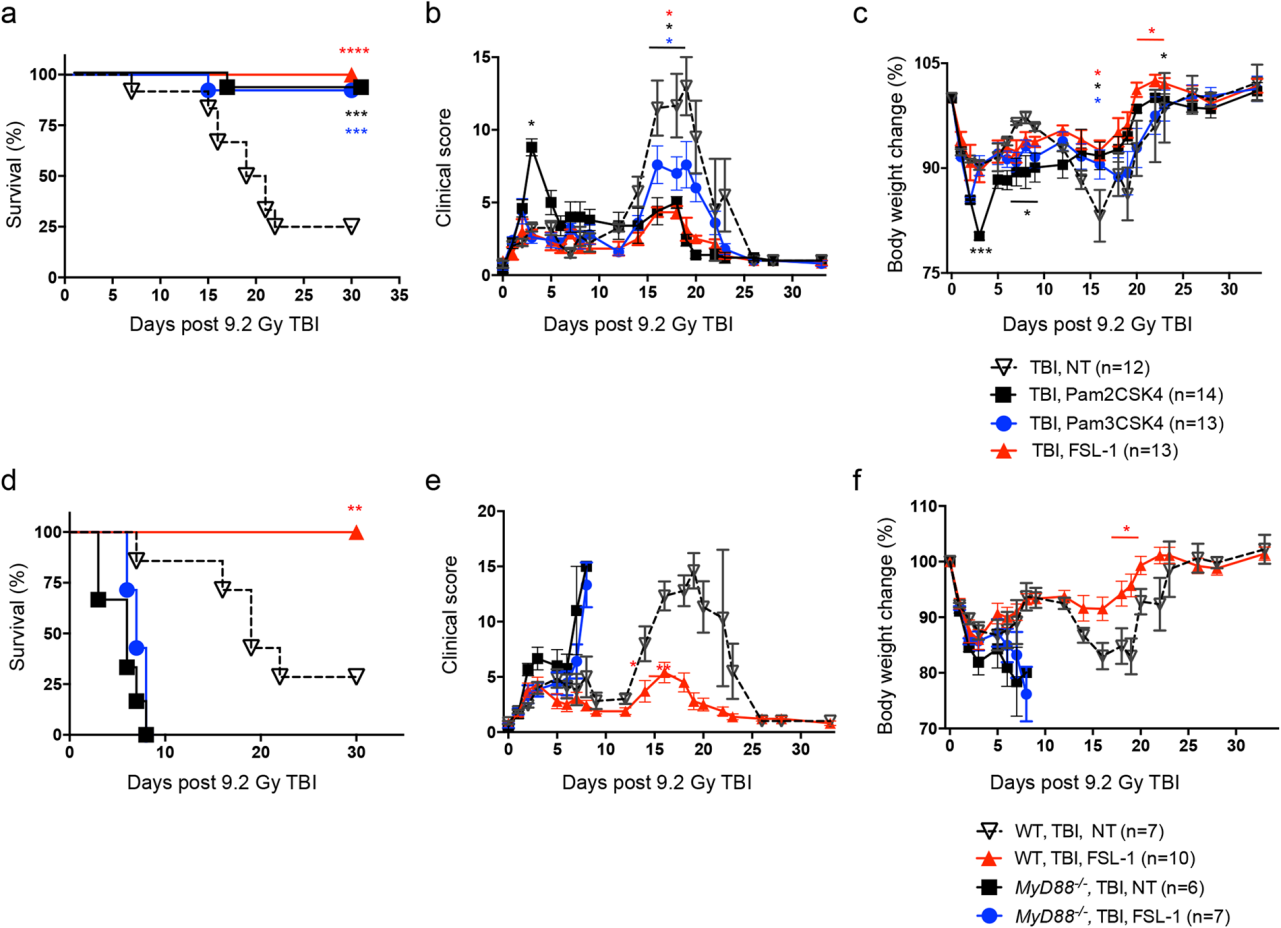
Radiomitigation is TLR2 and MyD88 specific. Male C57BL/6 mice were administered physiological water (NT), 0.25 mg/kg FSL–1, 0.25 mg/kg Pam2CSK4 or 0.25 mg/kg Pam3CSK4 at 24 hours after 9.2 Gy TBI exposure. (**a**) Survival was recorded through 30 days post TBI. Data is cumulative of 2 independent experiments with *n* = 4–6 mice per group per experiment. (**b**) Clinical score and (**c**) percent body weight change were assessed in comparison to irradiated, non–treated controls (TBI, NT). C57BL/6 or *MyD88* ^−/−^ male and female mice were administered physiological water (NT) or 0.25 mg/kg FSL–1 at 24 hours post 9.2 Gy TBI. (**d**) Survival, (**e**) clinical score, and (**f**) percent body weight change were assessed for 30 days post TBI. Data is representative of 2 experiments with *n* = 6 to 10 as indicated in legend. Data are shown as mean ± s.e.m. *P < 0.05, **P < 0.01, ***P < 0.001 or ****P < 0.0001 between treated and TBI, NT mice, with colors correlating to treatment group.

Several TLR2 ligands have been shown to exert protective and mitigative effects upon radiation exposure^19,22^. We further delineated radiomitigation activity of TLR2 ligands by irradiating male C57BL/6 mice and treating with either FSL–1 (TLR2/6 ligand), Pam2CSK4 (Pam2Cys–SKKKK, TLR2/6 ligand), or Pam3CSK4 (Pam3Cys–SKKKK, TLR1/2 ligand) at 24 hours post TBI. All three TLR2–activating ligands promoted survival (Fig. 2a), but showed differences in the well–being of treated animals. FSL–1 treatment resulted in lowered clinical scores and protection from weight loss as compared to control mice (Fig. 2b,c). Similarly, Pam2CSK4 and Pam3CSK4 treatment resulted in reduced clinical scores (Fig. 2b) and protected weight loss (Fig. 2c) as compared to control mice between 10 and 25 days after TBI. However, mice treated with Pam2CSK4, had an increased clinical score at day 3 (Fig. 2b), associated with marked weight loss as compared to control mice (Fig. 2c). A trend toward worsened outcome, especially at days 2 and 18, was also observed in Pam3CSK4–treated mice (Fig. 2c).

To confirm that activity of FSL–1 is MyD88–dependent, C57BL/6 and Myd88–deficient (*MyD88* ^−/−^) mice were exposed to 9.2 Gy TBI and 24 hours later, given FSL–1. All WT mice treated with FSL–1 survived for 30 days after TBI compared to 25% of non–treated WT mice (Fig. 2d). Irrespective of treatment, all *MyD88* ^−/−^ mice succumbed to radiation injury by 8 days post TBI (Fig. 2d). The lethality increase was associated with elevated clinical score and diminished weight (Fig. 2e,f). Actually, radiation injury (and lethality) appeared to be accelerated in *MyD88* ^−/−^ mice, as an increase in clinical score and decreased weight was not observed in non–treated WT mice until 2 weeks post TBI (Fig. 2e,f).

### FSL–1, a TLR2/6 ligand, shows beneficial radiomitigation properties

**Figure 3:**
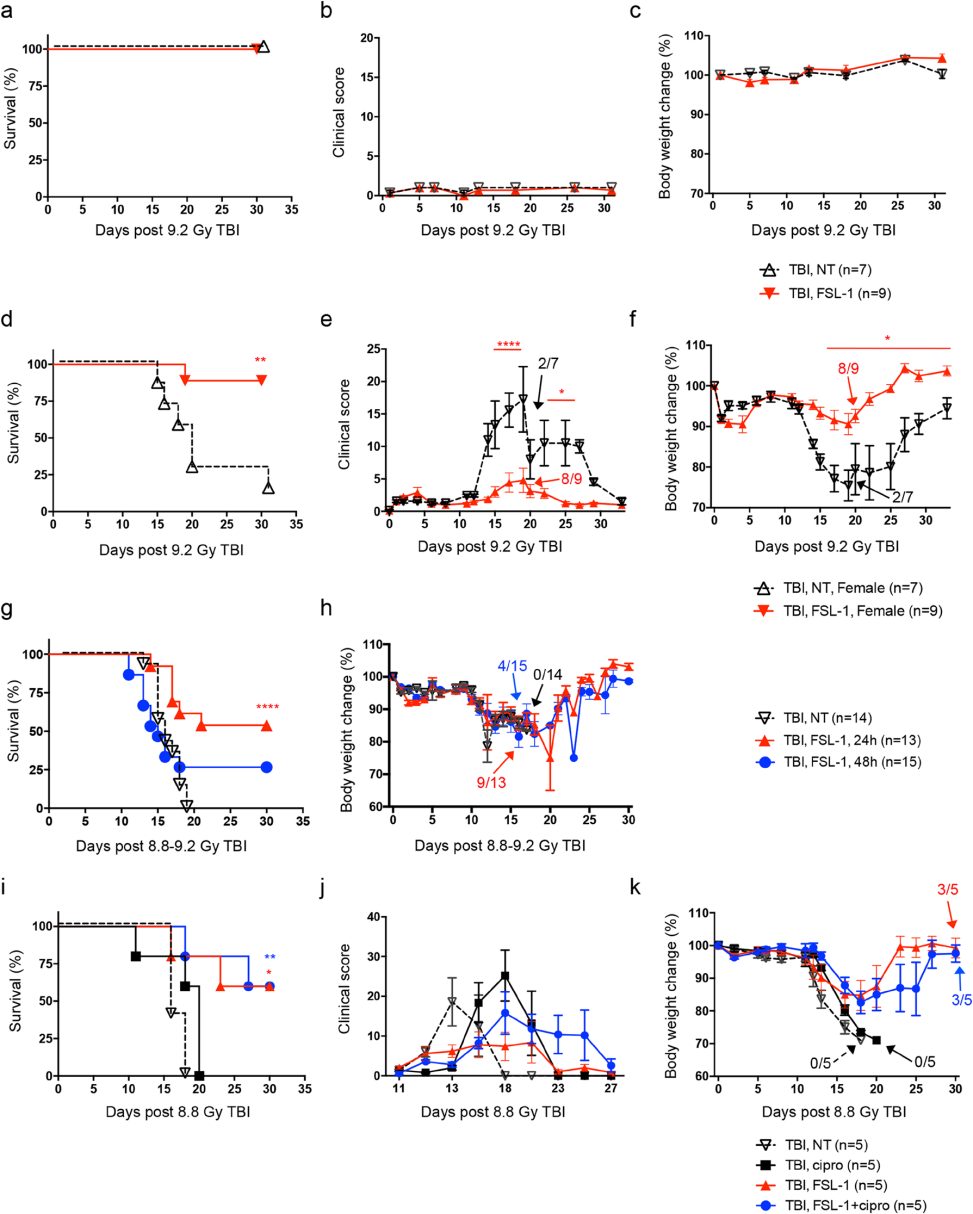
FSL–1, a TLR2/6 ligand, demonstrates radiomitigation properties. Toxicity was tested in male C57BL/6 mice, comparing physiologic water (NT) to 0.25 mg/kg FSL–1 treatment. (**a**) Survival, (**b**) clinical score, and (**c**) percent body weight change of mice were monitored over 30 days. Sex independency was tested by treating female C57BL/6 mice exposed to 9.2 Gy TBI with physiological water (NT) or 0.25 mg/kg FSL–1 given 24 hours post TBI. (**d**) Survival, (**e**) clinical score and (**f**) percent body weight change of female mice were assessed for 30 days. The effectiveness of FSL–1 treatment was compared by delivering physiological water (NT) or 0.25 mg/kg FSL–1 at 24 or 48 hours after 8.8 or 9.2 Gy TBI. (**g**) Survival and (**h**) percent body weight change were assessed for 30 days. Efficacy of FSL–1 combined with antibiotics treatment was evaluated by delivering physiological water (NT) or 0.25 mg/kg FSL–1 at 24 hours after 8.8 Gy TBI. On days 4 to 30 post TBI, ciprofloxacin was provided in autoclaved acidified water *ad libitum* from sipper tubes and also in wetted feed. Controls received no ciprofloxacin support, but did receive acidified water and moistened regular feed. (**i**) Survival, (**j**) clinical score and (**k**) percent body weight change were assessed for 30 days. Data (mean ± s.e.m.) are representative of 2 independent experiments with *n* = 5 to 15 as indicated in panel legends. *P < 0.05, **P < 0.01 or ****P < 0.0001 between treated and TBI, NT mice, with color representing treatment group. Arrows indicate time post TBI in which remaining mice in group began to recover.

The effectiveness of FSL–1 when given after radiation (radiomitigator) was examined by assessing consequences to drug treatment in absence of radiation, treatment of female mice after radiation and delayed administration post TBI. First, toxicity of FSL–1 was tested in the absence of TBI. FSL–1 treatment had no adverse effects on mouse survival, clinical score, or body weight when compared to naïve mice, suggesting minimal to no toxicity associated with FSL–1 under conditions for these studies (Fig. 3a–c).

To determine if FSL–1 radiomitigation is sex–independent, female C57BL/6 mice were administered FSL–1 at 24 hours post 9.2 Gy TBI. The 30 day survival of control females was 14.29% compared to 88.89% survival of females treated with FSL–1 (*P* = 0.002) (Fig. 3d). Survival of FSL–1–treated female mice was associated with drastically diminished clinical score (Fig. 3e). Furthermore, FSL–1–treated female mice maintained body weight 15 to 30 days post TBI, resulting in much less weight change compared to irradiated, non–treated female mice (Fig. 3f). This is the first comparison of male and female mice for TLR2–targeted radiomitigation studies.

To determine if FSL–1 treatment is effective when given later than 24 hours post TBI, C57BL/6 mice were administered FSL–1 at 48 hours after TBI. Although the 30 day survival of mice treated with FSL–1 at 48 hours post TBI was diminished compared to mice treated at 24 hours post TBI (Fig. 3g) (25% versus 50%, *P* = 0.0352), survival was still enhanced when comparing to mice receiving no treatment (Fig. 3g) (25% versus 0%, *P* = 0.1247). The capacity to evoke 25% survival rate is notable for targeting H–ARS, since most radiomitigators have not been examined at this delayed time or have failed when administered 48 hours after radiation^22^. Consistent with previous observations, no weight differences were observed between control and treated mice (Fig. 3h), with the caveat that all of the control mice succumbed to radiation by 2 weeks.

As ionizing radiation causes severe damage in both skin and gastrointestinal tract, the susceptibility of patients to systemic infection from endogenous and exogenous organisms prominently increases following radiation exposure^32^. Antibiotics treatment has been considered as an important component of any radiation mitigation strategy^33^. To examine whether FSL–1 synergizes with antibiotics to augment radioprotection, ciprofloxacin, a broad–spectrum fluoroquinolone previously tested in an acute radiation murine model^34^, was administrated to C57BL/6 mice or combined with FSL–1 treatment after animals were subjected to 8.8 Gy TBI. To avoid the added stress of daily injections and handling of irradiated mice, ciprofloxacin was provided *ad libitum* in the drinking water and as wetted feed, starting at day 4 and maintained throughout the 30 day monitoring period. Ciprofloxacin alone had a slight beneficial effect that was not statistically different from the profiles of survival, clinical score or weight change of animals receiving TBI with no treatment (Fig. 3i–k). The lack of a distinct effect of antibiotics may be partially due to the fact that these mice were housed in a specific pathogen–free environment. The 30 day survival of mice treated with FSL–1 combined with ciprofloxacin was similar to the survival profile of mice treated with FSL–1 only (Fig. 3i). Furthermore, there were no substantial differences in clinical score or body weight change between these two groups, which showed reduced clinical scores and rebounded weight maintenance (Fig. 3j,k). This suggests that FSL–1 administered in a single dose functions as an effective radiation countermeasures in the absence of antibiotics support.

### FSL–1 stimulates extramedullary hematopoiesis

**Figure 4:**
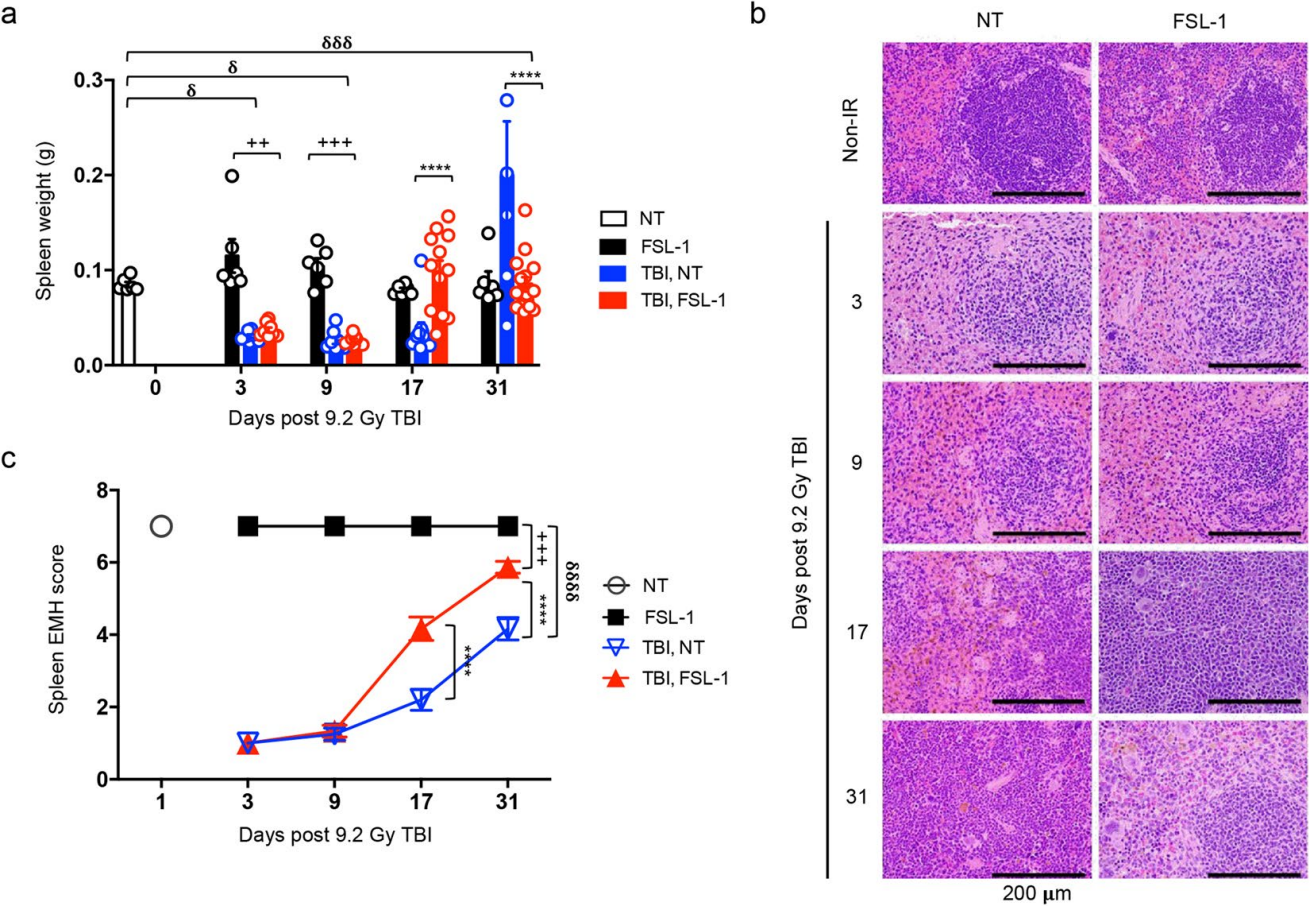
FSL–1 drives splenic EMH. Male C57BL/6 mice were administered physiological water (NT) or 0.25 mg/kg FSL–1 at 24 hours post 9.2 Gy TBI (*n* = 6–15 mice per group). (**a**) Spleens were harvested at 3, 9, 17 and 31 days after TBI, with weights shown. Each symbol represents one mouse. (**b**) Representative images of H&E stained spleen sections are shown. (**c**) Spleen EMH is quantified based on a scale described in Methods and represented as mean ± s.e.m. All data are representative of 3 independent experiments. ****P < 0.0001 between TBI, NT and TBI, FSL–1–treated mice. ^++^ *P* < 0.01 or ^+++^ *P* < 0.001 between TBI, FSL–1 and FSL–1–treated mice. ^δ^ *P* < 0.05, ^δδδ^ *P* < 0.001 or ^δδδδ^ *P* < 0.0001 between TBI, NT and NT control mice.

In mice, hematopoiesis is common in extramedullary sites (termed EMH), thereby allowing examination of H–ARS and recovery in bone marrow, peripheral blood, as well as in splenic parenchyma. First, we noted that spleens were distinctively smaller (indicative of H–ARS) in irradiated mice compared to non–irradiated mice at days 3 and 9 post TBI, regardless if also treated with FSL–1 (Fig. 4a; see Supplementary Fig. S1). At 17 days post TBI, spleens of FSL–1–treated irradiated mice showed recovery, as indicated by similar spleen sizes to spleens of non–irradiated mice (Fig. 4a; see Supplementary Fig. S1). Furthermore, spleens of FSL–1–treated, irradiated mice were appreciably heavier than non–treated, irradiated (TBI, NT) mice at 17 days (Fig. 4a). The size and appearance of spleens from FSL–1–treated mice remained similar to that of non–irradiated mice through 31 days following TBI, whereas the spleens of TBI, NT mice were radically enlarged at this later time point (Fig. 4a).

To examine how FSL–1 treatment contributes to EMH, hematologic features of spleens were examined. In non–irradiated mice, irrespective of FSL–1 treatment, splenic architecture was normal, with white pulp containing well–developed lymphocyte–rich follicles and red pulp containing venous sinusoids and scattered hematopoietic elements (Fig. 4b,c; see Supplementary Fig. S1). At 3 and 9 days post TBI, appreciable atrophy and lymphocyte depletion were observed in spleens of both non–treated and FSL–1–treated irradiated mice (Fig. 4b,c; see Supplementary Fig. S1). By 17 days post TBI, spleens of FSL–1–treated, irradiated mice had considerably more EMH than non–treated, irradiated mice, with splenic architecture approaching normal by day 31 in the FSL–1–treated mice (Fig. 4b,c; see Supplementary Fig. S1).

### FSL–1 enhances bone marrow hematopoiesis

**Figure 5:**
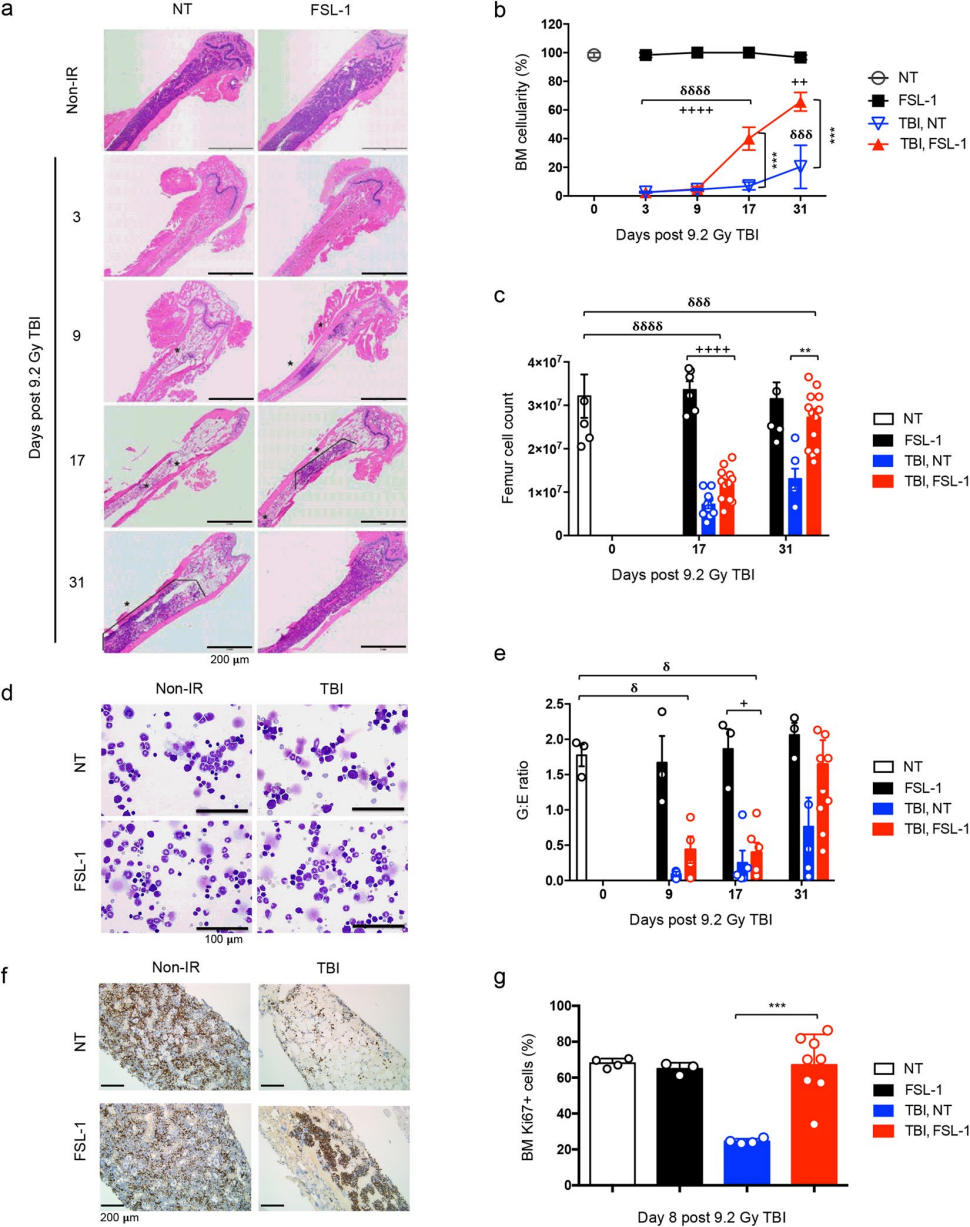
FSL–1 drives medullary hematopoiesis. Male C57BL/6 mice were given physiological water (NT) or FSL–1 at 24 hours post TBI. Femurs were collected at days 3, 9, 17 and 31 post TBI (*n* = 6–15 per group). (**a**) Representative images of H&E stained femur sections are shown. (**b**) Quantitative analysis of bone marrow (BM) percent cellularity and (**c**) BM cell counts per femur are presented. NT, non–radiated and not treated with FSL–1; FSL–1, non–radiated, but treated with FSL–1; TBI, NT, radiated and not treated with FSL–1; TBI, FSL–1, radiated and treated with FSL–1. (**d**) Representative modified Wright stained cytology images are shown. (**e**) BM cellular differentiation is represented as ratio of Granulocytic to Erythroid cells (G:E ratio). (**f**) Representative images of Ki67 stained femur sections are shown. (**g**) Quantitative analysis of Ki67 positive cells (% of total cells) is summarized from 3 random fields of view. The data includes 713 cells counted for NT samples, 1370 cells counted for FSL–1–treated samples, 858 cells counted for TBI, NT samples and 1979 cells counted for TBI, FSL–1 samples. Data are representative of 3 independent experiments. Graphed data are mean ± s.e.m. **P < 0.01 or ***P < 0.001 between TBI, NT and TBI, FSL–1–treated mice. ^+^ *P* < 0.05, ^++^ *P* < 0.01 or ^++++^ *P* < 0.0001 between TBI, FSL–1–treated and FSL–1 only treated mice. ^δ^ *P* < 0.05, ^δδδ^ *P* < 0.001 or ^δδδδ^ *P* < 0.0001 between TBI, NT and NT control mice. In c, e and g, each symbol represents one mouse.

Given increased EMH evident in spleens, we then explored the effect of FSL–1 on bone marrow (BM). Regardless of drug treatment, femurs of non–irradiated mice were normal in appearance (95–100% cellularity; Fig. 5a,b; see Supplementary Fig. S2). Consistent with radiation injury resulting in massively reduced cellularity, extensive stromal injury and cell death were observed in BM from non–treated and FSL–1–treated mice 3 days post TBI (Fig. 5a,b; see Supplementary Fig. S2). By day 9 post TBI, repopulation of viable adipocytes, indicative of repair processes, was observed in the BM of non–treated and FSL–1–treated mice, along with repopulation of hematopoietic cells in FSL–1–treated mice only (Fig. 5a; see Supplementary Fig. S2). By 17 days post TBI, mean BM cellularity in FSL–1–treated irradiated mice was notably increased compared to non–treated, irradiated mice (Fig. 5a,b). By 31 days post TBI, BM cellularity of FSL–1–treated mice ranged from 50–90%, which was considerably elevated compared to non–treated, irradiated mice (Fig. 5a,b). Femur cell counts mirrored the phenotype observed in the histologic samples, with femoral cell numbers of FSL–1–treated irradiated mice rebounding to numbers found in non–irradiated mice (Fig. 5c) with a similar kinetic profile.

To further characterize hematopoiesis in the femurs of irradiated mice, cytologic preparations were evaluated for the relative proportion of granulocytic and erythroid lineages (G:E ratio) (Fig. 5d,e). A normal G:E ratio is approximately 2, as observed in non–irradiated mice independent of FSL–1 treatment (Fig. 5d,e). The G:E ratio was appreciably diminished by 9 and 17 days following TBI in non–treated and FSL–1–treated mice, consistent with the earlier recovery of the erythroid lineage (Fig. 5e). As depicted in the representative cytology image, an increased G:E ratio in the femurs of FSL–1–treated, irradiated mice was observed by day 31 post TBI, whereas a diminished G:E ratio persisted in irradiated, but non–treated mice (Fig. 5d,e).

To further investigate BM cell regeneration, Ki67 staining was conducted on femur samples and proliferating Ki67+ cells were tabulated. In the absence of radiation, FSL–1 treatment showed no influence on BM cell proliferation as compared with the non–treated group (Fig. 5f,g). By 8 days post TBI, in the absence of FSL–1 radiomitigation, massive cell death and scarce cell proliferation were found in BM samples. On the other hand, FSL–1 treatment effectively supported the recovery of Ki67+ cells to a level that was not statistically different from what was found in the non–irradiation groups (Fig. 5f,g). Taken together, these results suggest that FSL–1 dramatically enhances cellular proliferation that contributes to the accelerated regeneration of bone marrow cells.

### FSL–1 promotes peripheral blood recovery

**Figure 6:**
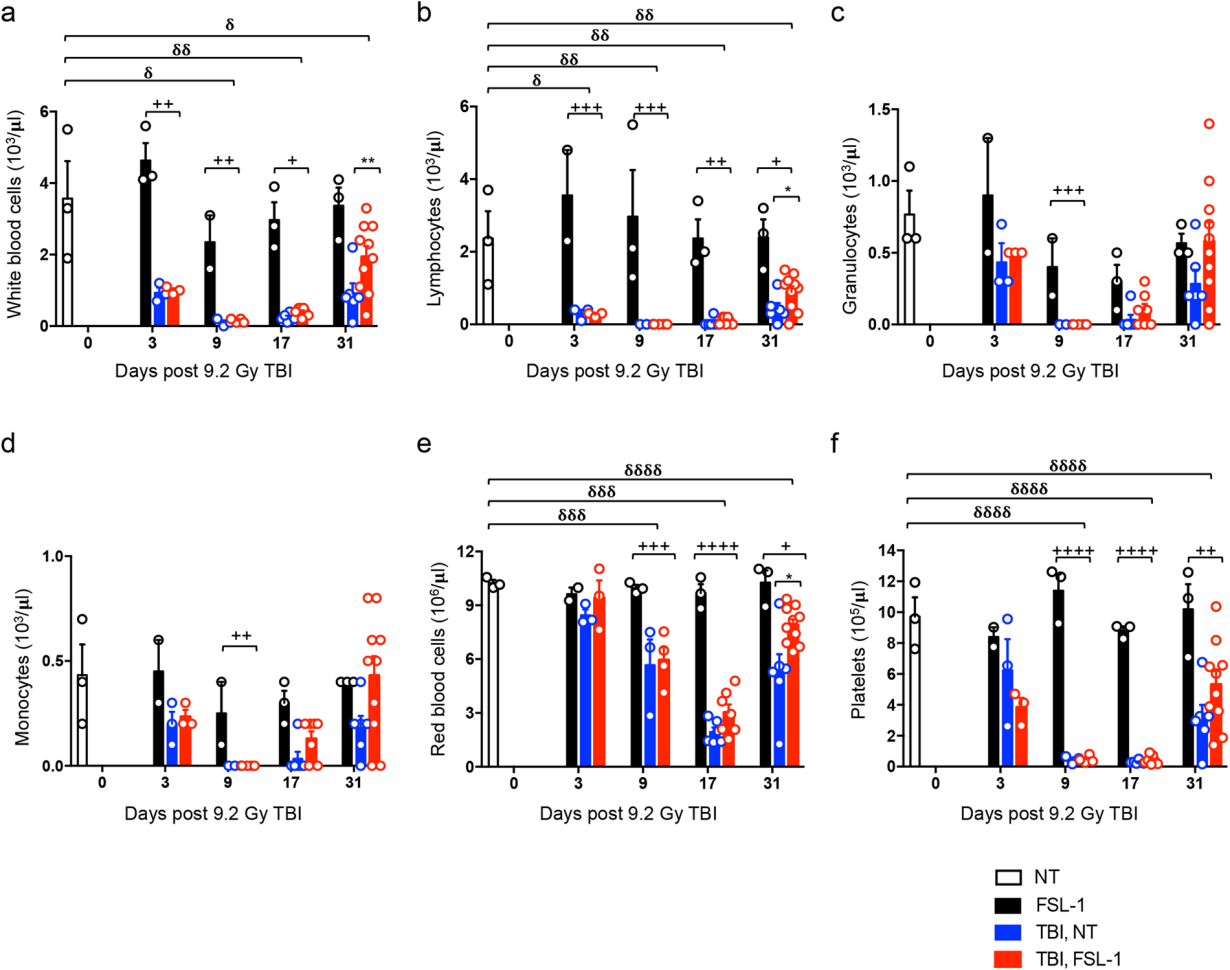
FSL–1 enhances peripheral blood repopulation. Male C57BL/6 mice were administered physiological water (NT) or FSL–1 at 24 hours post 9.2 Gy TBI. Peripheral blood was collected with (**a**) white blood cell, (**b**) lymphocyte, (**c**) granulocyte, (**d**) monocyte, (**e**) red blood cell and (**f**) platelet populations counted. The number of mice examined are: *n* = 3 for NT and FSL–1 only groups; *n* = 6 for TBI, NT mice; *n* = 6–9 (days 3 and 9); *n* = 7 (day 17) and *n* = 10 (day 31) for TBI, FSL–1–treated mice. Data are represented by mean ± s.e.m., and are tallied from 2–3 independent experiments. Each symbol represents one mouse. *P < 0.05 or **P < 0.01 between TBI, NT and TBI, FSL–1–treated mice. ^+^ *P* < 0.05, ^++^ *P* < 0.01, ^+++^ *P* < 0.001 or ^++++^ *P* < 0.0001 between TBI, FSL–1 and FSL–1 only treated mice. ^δ^ *P* < 0.05, ^δδ^ *P* < 0.01, ^δδδ^ *P* < 0.001 or ^δδδδ^ *P* < 0.0001 between TBI, NT and NT control mice.

In addition to BM depletion, consequences of radiation can manifest as cytopenia in peripheral blood. Previously, administration of the TLR2 ligand CBLB613, a day prior to radiation, resulted in the enhancement of peripheral blood cellularity within the first 48 hours following TBI^19^; however, no other time points were examined to determine if this increase was achievable with post–radiation dosing. Here, FSL–1 treatment alone did not alter white blood cell (WBC) or red blood cell (RBC) populations in the periphery of non–irradiated C57BL/6 mice (Fig. 6). To assess the effects of post–radiation FSL–1 treatment on cytopenia, peripheral blood samples were analyzed at days 3, 9, 17 and 31 after 9.2 Gy TBI. We found that radiation caused partial to complete depletion of all blood cell populations through 17 days after TBI, without any protection by FSL–1 treatment (Fig. 6). By 31 days post TBI, WBC (granulocyte, monocyte) and platelet populations were nearly fully or partially restored to baseline levels in FSL–1–treated, irradiated mice as compared to non–treated mice (Fig. 6a,c,d,f). Furthermore, RBCs were elevated in FSL–1–treated, irradiated mice at this later time as compared to non–treated, irradiated mice (Fig. 6e). These data point to the promotion of peripheral blood cell recovery with FSL–1 treatment after radiation.

### FSL–1–induced G–CSF correlates with enhanced hematopoiesis

**Figure 7:**
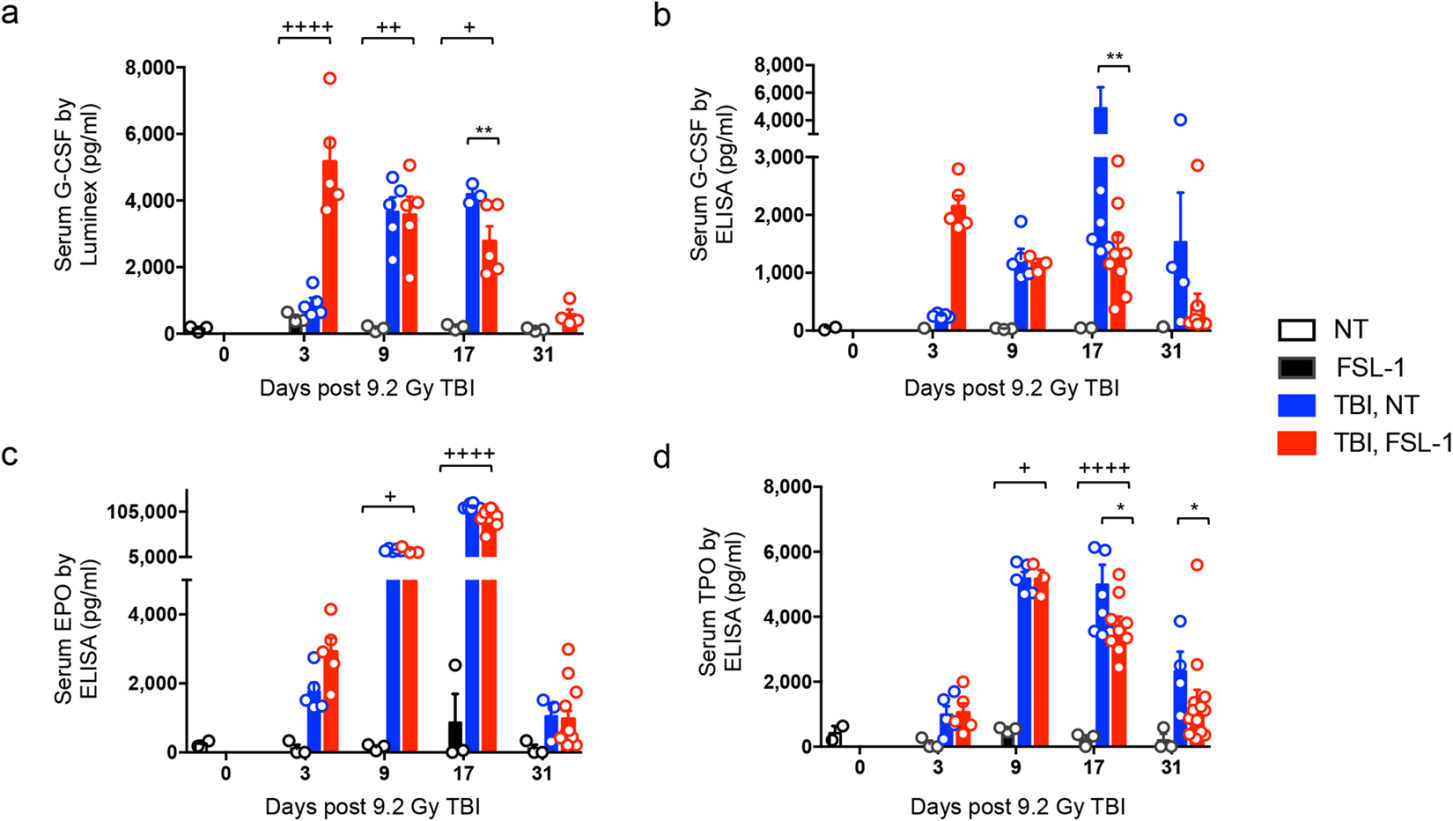
G–CSF biomarker is elevated early in FSL–1–treated mice. Male C57BL/6 mice were administered physiological water (NT) or FSL–1 at 24 hours post 9.2 Gy TBI. Peripheral blood was collected on days 3, 9, 17 and 31 post TBI. G–CSF levels were assessed by (**a**) luminex or (**b**) ELISA. (**c**) EPO and (**d**) TPO levels were assessed by ELISA. Luminex data represents one experiment, while ELISA data are cumulative of 2 independent experiments. Each symbol represents one mouse. *P < 0.05 or **P < 0.01 between TBI, NT and TBI, FSL–1–treated mice. ^+^ *P* < 0.05, ^++^ *P* < 0.01 or ^++++^ *P* < 0.0001 between FSL–1 and TBI, FSL–1–treated mice.

Early response biomarkers such as hematopoietic proteins and blood cell counts have been used as indicators of radiation exposure in order to assess severity of dose and efficacy of treatments^35,36^. To determine if enhanced H–ARS recovery in FSL–1–treated mice is due to cooperation between TLR2/6 stimulation and growth factors, serum factors were measured at 3, 9, 17 and 31 days post TBI plus or minus FSL–1 treatment. Using two methods of detection, we found that FSL–1 caused a shift in the kinetics of serum G–CSF. Specifically, G–CSF was dramatically elevated in FSL–1–treated, irradiated mice at day 3 post TBI, followed by a steady decline observed through day 31 after TBI, when levels were similar to that of non–irradiated mice (Fig. 7a,b). Conversely, G–CSF was low at day 3, detectable at day 9 and peaked later by day 17 in irradiated mice that were not treated with FSL–1 (Fig. 7a,b). Thus, FSL–1 induced a drastically elevated level of G–CSF soon after TBI (day 3 post exposure) when compared to non–treated, irradiated controls. This is consistent with higher levels of hematopoiesis observed with FSL–1 treatment (as shown in Figs 4–5). We examined other serum biomarkers by luminex analysis, but did not find any notable correlations with FSL–1 treatment and/or radiation (see Supplementary Table S2), except for FSL–1–induced serum LIX/Cxcl5 or granulocyte chemotactic protein 2, which is involved in the mobilization of hematopoietic stem cells to the periphery^37^. Also, delayed elevation in EPO and TPO was evident by 9 and 17 days after irradiation in both non–treated and FSL–1–treated samples. FSL–1 treatment did not alter EPO or TPO levels, except for slight reductions in TPO at days 17 and 31 after TBI (Fig. 7c,d). In summary, these data indicate that FSL–1 supports hematopoietic recovery through the induction of stimulating factors, such as G–CSF.

## Discussion

The results in this report show that ligands for TLR2/6, but not for TLR4, 5 nor 9, promoted survival when administered at 24 hours following TBI. Variability in protection with other TLR ligands compared to previously published work is likely due to ligand specificity (i.e. Flagellin FliC used here versus CBLB502), time of administration, mouse strain and/or radiation source^13,17,18^ as well as tissue expression patterns of effector receptors. However, head–to–head comparison indicates that FSL–1 confers considerably greater protection with a dearth of associated adverse effects. FSL–1 provides substantial protection from radiation injury when administered 24 hours post TBI. Although only modest radiomitigation remains when FSL–1 was administered at 48 hours (25% survival), this level of protection is still notably greater than other published countermeasures when administered at this delayed time after irradiation^22^. Importantly, effectiveness of FSL–1 as a radiomitigator is sex–independent.

It is well understood that MyD88–dependent signaling is crucial for recovery following radiation^21,22,31,38^. Furthermore, it has been shown that TLR ligands administered prior to radiation mediate effects through MyD88–dependent mechanisms. Similarly, data herein show that the effects of FSL–1 are MyD88–dependent. Independent of treatment with FSL–1, *MyD88* ^−/−^ mice succumb to radiation injury by 10 days post exposure. Moreover, death in *MyD88* ^−/−^ mice is substantially accelerated as compared to WT controls treated with FSL–1.

Data herein demonstrate that FSL–1 treatment drives hematopoiesis in medullary and extramedullary sites, thereby enhancing recovery following sub–lethal radiation. Given that circulating G–CSF levels are elevated in the periphery of FSL–1–treated mice as early as 3 days post TBI, TLR2 stimulation may accelerate hematopoiesis in a G–CSF–dependent manner. This model is consistent with previous work noting G–CSF as a biomarker for radioprotection with the TLR5 agonist, CBLB502^25^. G–CSF is a growth factor that drives granulocyte and stem cell production within the BM, as well as the recruitment of cells into the periphery. Collaboration can also occur between G–CSF and other hematopoietic growth factors to impact lymphoid and erythroid cell development and functions. Recent studies suggest both a synergistic and antagonistic relationship between TLR2 ligands and G–CSF in the regulation of hematopoiesis^10,39,40^. While FSL–1 administered post TBI is associated with increased G–CSF secretion and accelerated hematopoiesis, delayed enhancement of G–CSF found within serum of non–treated, irradiated mice is likely a compensatory mechanism to restore severe lymphatic tissue damage.

Beyond the use for accidental nuclear radiation exposure, FSL–1 treatment should be considered for application alone or in combination with cancer–related radio– or chemotherapies. G–CSF has been approved by the U.S. FDA for the prevention of febrile neutropenia in cancer patients who receive myelosuppressive chemotherapy. It will be of interest to test FSL–1 in cancer models for determining its potential application in cancer therapy. There is substantial research demonstrating the benefits of G–CSF in protecting individuals from adverse effects, including insults to the hematopoietic system associated with radio– or chemotherapies^41,42^. Specifically, G–CSF contributes to the mobilization of hematopoietic cells in cancer patients undergoing various treatments^43^. We suggest that FSL–1–driven TLR2/6 stimulation can promote increased G–CSF, therefore resulting in enhanced hematopoietic cell mobilization and regeneration. This mechanism has been observed in the intestine where TLR2 stimulation drives monocyte differentiation in a G–CSF–dependent manner^39^. In a model of graft versus host disease, it was demonstrated that G–CSF mobilization of myeloid cells is diminished in *Tlr2* ^−/−^ mice and in mice treated with TLR2 inhibitors^40^. Our radiation exposure data suggest that TLR2/6 stimulation by FSL–1 drives G–CSF, which then mediates hematopoietic cell mobilization.

Although G–CSF treatments provide substantial therapeutic benefits, several studies have raised concerns about the safety of this growth factor^33,44–46^. One study raised the concern of increased long term risks for developing myelodysplasia (MDS) and acute myeloid leukemia (AML) in stem cell donors who receive repeated G–CSF dosing for cell mobilization^47^. Another study reported a trend toward increased incidence of MDS/AML in breast cancer patients receiving G–CSF support with chemotherapy treatment^48^. Lastly, severe congenital neutropenia patients who were treated with higher doses of G–CSF showed a more than 3–fold increased risk of transformation to MDS/AML compared with those who received lower doses^49,50^. In contrast in our experiments, a subset of mice that received FSL–1 treatment after total body radiation have remained alive for more than 600 days, without the presentation of diseases or cancers. Furthermore, we performed Magnetic Resonance Imaging (MRI) to analyze these long term survivors and found no physiologic changes in the brain, gut, kidney or spleen (see Supplementary Fig. S3). These observations provide impetus to suggest that FSL–1 may be a safer treatment compared to G–CSF.

In 2015, G–CSF was approved as a drug by the U.S. FDA for treating radiation–induced hematopoietic damage. It has also been approved by the Centers for Disease Control and Prevention for administration to victims exposed to a radiological nuclear incident. The radio–protective efficacy of G–CSF is dependent on drug dose as well as drug treatment schedule. Shakhov *et al*. showed G–CSF or Neupogen® (Amgen, Inc.) increased the survival of irradiated mice only when injected subcutaneously daily from days 1 to 16 after 7.96 Gy irradiation^22^. In our study, a single dose of FSL–1 given 24 hours post–radiation provided as high as 80% protection, which suggests FSL–1 may be superior in convenience and effectiveness as a radiation mitigator. Therefore, by extension, it is possible that FSL–1 may provide greater potential in comparison to the commonly used G–CSF treatment in cancer patients undergoing radio– and/or chemotherapy treatment.

## Methods

### Animals

Eight to twelve week old mice were used for all studies. C57BL/6 mice (WT, Jackson Laboratories, Bar Harbor, ME) and *MyD88* ^−/−^ mice (originally from Dr. Shizou Akira, Osaka University, Osaka, Japan) were bred under pathogen–free conditions at the University of North Carolina Chapel Hill (UNC–CH) facilities accredited by the Association for the Assessment and Accreditation of Laboratory of Animal Care. For some studies, 6 week old male C57BL/6 mice were ordered from Jackson Laboratories and allowed 2–4 weeks of acclimation prior to experimentation. All protocols were established based on the Guide for the Care and Use of Laboratory Animals (2011) and approved by the UNC–CH Institutional Animal Care and Use Committee.

### Total body irradiation

Total body irradiation (TBI) of mice was performed using an attenuator (X–302) with a ^137^Cesium gamma–ray irradiating source (Mark I, Model 68–1, J.L. Shepard & Associates, San Fernando, CA). Alternatively, a Gammacell® 40 Exactor ^137^Cesium source (Serial no. 265, Best Theratronics, Ottawa, Ontario) was used for γ–irradiation, with dosimetry variation of +/− 0.07 Gy as determined with phantoms by Dr. Ke Sheng, UCLA, Los Angeles, CA. For TBI, non–anesthetized mice were placed in ventilated plastic pie cages and exposed to 8.8 Gy or 9.2 Gy TBI. Following TBI, mice were housed in sterile autoclaved cages and provided standard chow and water *ad libitum* unless otherwise noted.

Mice were monitored for changes in body weight, surface body temperature and body parameters through 30 days post TBI unless otherwise noted. A clinical score was determined using a cumulative scoring system (see Supplementary Table S1) based on weight loss, temperature change, physical appearance, posture, mobility, food consumption and hydration^51^. Immediate indications for euthanasia included: weight loss greater than 25% of starting body weight, unconsciousness, an inability to remain upright, agonal respiration (gasping) or convulsions.

### TLR ligand administration

All TLR ligands (InvivoGen, San Diego, CA) were resuspended in sterile, vaccination–grade physiological water. Twenty–four hours following TBI, mice were given an i.p. injection of physiological water (no treatment, NT), 2.5 mg/kg CpG–ODN 2395 (tlrl–2395), 2.0 mg/kg Flagellin FliC (vac–fla), 0.25 mg/kg MPLA (tlrl–mpla), 0.25 mg/kg FSL–1 (tlrl–fsl), 0.25 mg/kg Pam2CSK4 (tlrl–pm2s–1) or 0.25 mg/kg Pam3CSK4 (tlrl–pms). The total volume administered per mouse ranged from 50–70 μl based on the weight of the mouse and ligand concentration.

### Antibiotics Treatment

Male C57BL/6 mice received 8.8 Gy TBI and 24 hours later the mice were given an i.p. injection of physiological water (NT) or 0.25 mg/kg FSL–1. On days 4 to 30 post TBI, 0.67 mg/ml ciprofloxacin (Sigma, catalog 17850) was provided in autoclaved acidified water *ad libitum* from sipper tubes and also as wetted feed in Petri dishes set on the cage bottom. Controls received no ciprofloxacin, but did receive autoclaved acidified water plus wetted feed on days 4 to 30 after radiation exposure. Mice were monitored for changes in body weight, surface body temperature and other body parameters through 30 days post TBI as described above.

### Histopathology and cytology

To assess tissue pathology, isolated spleens and femurs were fixed in 10% neutral–buffered formalin, paraffin–embedded, and sectioned (4 micron thickness) at the UNC Lineberger Animal Histopathology Core Facility. Prior to paraffin–embedding, femurs underwent an additional decalcification step in Immunocal^TM^ (StatLab, McKinney, TX). Slides were then stained with hematoxylin and eosin (H&E).

Slides of femur sections were also stained with Ki67 antibody (D3B5, Cell Signaling, 12202) to examine proliferative capacity. Ki67 positive cells were quantified using ImageJ (Version 2.0.0–rc–46/1.50 g). Three random fields of view were examined. Percent of Ki67^+^ cells was tabulated as follows: Ki67+ DAPI+ cells/total cells per view of femur section, with more than 700 cells counted for each cohort.

Histologic and cytologic samples were scored by a Board Certified Pathologist (NDM) who was blinded to experimental conditions. Scoring of spleen histology and BM cytology were performed using an Olympus BX51 microscope with Olympus objective lenses: PLAPON 2× (numerical aperture, na = 0.08, overall original magnification 20×), UPlanFL N 4× (na = 0.13, magnification 40×), and UPlanFL N 60× (na = 1.25 Oil, magnification 600×). Images were taken with a DP71 camera and DPController v3.3.1.292 software (Olympus, Center Valley, PA). Scoring of BM histology was performed using an Olympus BX43 microscope with Olympus objective lenses PLAPON 2× (na = 0.08, magnification 20×) and UPlanFL N 4× (na = 0.13, magnification 40×). Images were taken with a DP27 camera and CellSens Dimension 1.13 software (Olympus). For all microscopic studies, evaluation was performed at ambient temperature. Image processing was limited to contrast adjustment and sharpening, and was performed using Adobe Photoshop CS4 v11.0.2 (Adobe, San Jose, CA).

To detail changes in spleen histology, a semi–quantitative scoring system was developed, reflecting sequential histologic features observed in recovering spleens from irradiated animals. In general, this scoring system was based on the amount and patterns of extramedullary hematopoiesis (EMH) as described here: 1, atrophy; 2, atrophic white pulp with less than 20% EMH; 3, atrophic white pulp with 20–60% EMH; 4, atrophic white pulp with 60–100% EMH; 5, early white pulp recovery with extensive EMH in red pulp; 6, well–developed white pulp with extensive EMH in red pulp; 7, normal spleen.

H&E stained femur sections were scored based on the overall percentage of marrow space occupied by hematopoietic cells as compared to adipocytes and expressed as percent bone marrow (BM) cellularity [(marrow space occupied by hematopoietic cells/total marrow space) ×100%]. For cytologic analysis of BM, femurs were flushed with Alpha–MEM media containing 10% FBS and 100 × Pen/Strept. BM cells were homogenized to obtain a single cell suspension, and then serially diluted to 1:1, 1:2 and 1:4 ratios. Cytospin slides were prepared by centrifugation for 5 minutes at 1,000 × *g*, fixed in methanol and stained using a modified Wright staining protocol. A manual differential count of 100 cells on each slide was performed, and the ratio of granulocytic to erythroid lineage cells was calculated.

### Peripheral blood assessment

Whole blood was collected by cardiac puncture and placed in EDTA–coated tubes. Standard hematological tests to examine WBC and RBC populations were performed by the UNC–CH Animal Clinical Chemistry Facility using an Animal Blood Counter (Heska, Loveland, CO).

### Cytokine analysis

Whole blood was collected by cardiac puncture and centrifuged to isolate serum. Serum samples were analyzed by Enzyme–Linked Immunosorbent Assay (ELISA) (G–CSF; MCS00, R&D Systems, Minneapolis, MN) or by multiplex analyte assay using Luminex technology (EMD Millipore, Darmstadt, Germany) according to manufacturers’ protocols.

### Statistics

All data are represented as mean ± s.e.m. Significance between multiple groups was determined using one–way analysis of variance (ANOVA) with the Tukey–Kramer post hoc test for multiple comparisons. Statistical significance of survival curves was determined using Kaplan and Meier analysis. A *P*–value < 0.05 was considered statistically significant. All statistical analyses were performed using Prism (GraphPad Software Inc, La Jolla, CA).

### Data availability

All data generated or analyzed during this study are included in this article (and its Supplementary Information file).

## Electronic supplementary material

Supplementary Information
