# Supplementary material for: The Toll–Like Receptor 2/6 Agonist, FSL–1 Lipopeptide, Therapeutically Mitigates Acute Radiation Syndrome
Source: Sci Rep. 2017 Dec 11;7:17355. doi: 10.1038/s41598-017-17729-9 (PMC5725477; doi:10.1038/s41598-017-17729-9)
Supplement: Supplementary file 1 — Supplementary Information [file 41598_2017_17729_MOESM1_ESM.pdf]

Supplementary Information

The Toll-Like Receptor 2/6 Agonist, FSL-1 Lipopeptide, Therapeutically Mitigates Acute Radiation Syndrome

Cathryn J. Kurkjian, Hao Guo, Nathan D. Montgomery, Ning Cheng, Hong Yuan, Joseph R. Merrill, Gregory D. Sempowski, W. June Brickey, and Jenny P.-Y. Ting

Supplementary Methods

Magnetic resonance imaging. MR imaging study was conducted on a 9.4T animal MRI scanner (BioSpec 94/30 model, Bruker BioSpin Inc) in the Small Animal Imaging Facility. Anatomical T2-weighted MR images on brains were acquired using the Rapid Acquisition with Relaxation Enhancement (RARE) sequence with the following acquisition parameters: Echo time (TE) of 30ms and Repetition time (TR) of 3323 ms, 0.5 mm slice thickness, 200x200 matrix size, and 100x100 micron in-plane resolution. For MRI on abdominal regions, gated T2-weighted MRI was acquired using the RARE sequence and the following parameters: TE/TR of 21/4000 ms, 1 mm slice thickness, 192x256 matrix size, and 130x117 micron in-plane resolution. Mice were maintained under anesthesia using isoflurane (1.5%) mixed with oxygen during MR scanning and recovered from anesthesia after imaging.

Supplementary Table S1. Clinical Score Parameters.

Category	Score	Description
Body weight[a]	0	normal (<5% change from initial weight)
	1	5-10% weight change
	2	10-14.9% weight change
	3	15-19.9% weight change
	4	20-24.9% weight change
	6	≥25% weight change
Body temperature[b]	0	normal (33-35°C)
	2	30-32.9^{o}C
	4	28-29.9^{o}C
	6	<28°C
Hydration	0	normal
	1	mildly dehydrated (<1sec skin tent)
	2	moderately dehydrated (1-2sec skin tent)[c]
	4	severely dehydrated (>2sec skin tent)[c]
Appearance	0	normal
	1	lack of grooming
	2	rough hair/coat
	3	very rough hair/coat
Posture	0	normal
	1	sitting in hunched position
	4	hunched posture, head resting on floor
	6	lying prone on cage floor
Activity	0	normal
	1	reduced/minor changes in behavior
	3	changes in activity and respiratory rate or effort
	6	moves only when stimulated
Appetite	0	normal
	1	reduced
	2	not eating since last daily check point
	3	not eating for last 2 daily check points

[a], assessed weekly, then every other day when 10% weight change reached, and daily after 15% weight change reached.

[b], ventral surface temperature determined using infrared thermometer.

[c], with supplemental fluids given s.c. and hydration such as hydrogel provided.

Endpoint for euthanasia: any single parameter score of 6 or combined score of 15.

Supplementary Table S2. Serum factors induced after TBI with or without FSL-1 treatment.

		Amount (pg/ml ± s.e.m.)			
Serum factor (pg/ml threshold detection)	Treatment	Day 3	Day 9	Day 17	Day 31
M-CSF (1.5)	NT	ND	ND	ND	2.7 ± 1.2
	FSL-1	1.5	1.5	2.4 ± 0.9	1.5
	TBI, NT	4.3 ± 1.0	2.2 ± 0.7	6.2 ± 2.0	ND
	TBI, FSL-1	5.0 ± 2.7	12.9 ± 10.6	1.5	1.5
IL-5 (1.6)	NT	ND	ND	ND	6.2 ± 3.2
	FSL-1	1.6	1.6	4.7 ± 2.5	4.9 ± 3.3
	TBI, NT	267.2 ± 237	75.1 ± 17.2	26.9 ± 7.9	ND
	TBI, FSL-1	23.0 ± 3.4	57.1 ± 7.6	24.3 ± 4.9	27.1 ± 14.7
IL-6 (1.6)	NT	ND	ND	ND	1.6
	FSL-1	3.9 ± 2.3	1.6	1.6	1.6
	TBI, NT	294 ± 291.1	6.4 ± 2.0	202.2 ± 193	ND
	TBI, FSL-1	3.8 ± 0.9	4.4 ± 1.8	7.2 ± 1.8	2.1 ± 0.5
IL-9 (44.1)	NT	ND	ND	ND	44.1
	FSL-1	139.4 ± 52	125 ± 59.6	229.5 ±185	83.8 ± 39.7
	TBI, NT	368 ± 196.9	59.9 ± 15.8	58.8 ± 14.7	ND
	TBI, FSL-1	2176 ±1957	205 ± 66.7	44.1	44.1
IL-13 (1.4)	NT	ND	ND	ND	11 ± 4
	FSL-1	19.9 ± 1.2	10.6 ± 3.6	20.3 ± 2.6	13 ± 3
	TBI, NT	933 ± 919	7 ± 0	17.1 ± 5.6	ND
	TBI, FSL-1	19.8 ± 8.9	11.0 ± 2.5	7 ± 0	11.4 ± 2.8
Cxcl1/KC (1.6)	NT	ND	ND	ND	108 ± 24.7
	FSL-1	246.5 ± 5	61.6 ± 16.3	90.6 ± 6.0	68 ± 20.8
	TBI	341 ± 170.4	150 ± 24.8	1231 ± 1148	ND
	TBI, FSL-1	351.1 ± 22	171 ± 37.9	124.8 ± 9.2	163.5 ± 57.8
Cxcl2/MIP-2 (6.6)	NT	ND	ND	ND	75.1 ± 12.2
	FSL-1	76.7 ± 2.4	47.6 ± 9.1	58.7 ± 19.8	38.1 ± 12.8
	TBI, NT	262 ± 170.4	41.8 ± 9.6	338 ± 262.2	ND
	TBI, FSL-1	101.3 ± 11	78.9 ± 42.7	35.8 ± 10.6	35.3 ± 12.0
Cxcl5/LIX (180.4)	NT	ND	ND	ND	9353 ± 518
	FSL-1	4501 ± 581	4251 ±839*	6494 ±2206†	6077 ± 1640†
	TBI, NT	3225 ± 1151	180.4	180.4	ND
	TBI, FSL-1	4352 ± 1166	180.4	180.4	784.7 ± 232
Cxcl9/MIG (9.0)	NT	ND	ND	ND	67.7 ± 27.6
	FSL-1	89.9 ± 7.8	118 ± 10.8	110.1 ± 3.3	52.6 ± 3.3
	TBI, NT	215.3 ± 126	83.4 ± 9.4	44.5 ± 5.5	ND
	TBI, FSL-1	71.5 ± 7.5	112.8 ± 57	79.1 ± 12.4	166.4 ± 32.6

Cxcl10/IP-10 (1.6)	NT	ND	ND	ND	146.2 ± 27.2
	FSL-1	245 ± 11	200.6 ± 22	182.4 ± 37.7	171.8 ± 38.6
	TBI, NT	495.7 ± 275	156 ± 24.5	194.1 ± 64.5	ND
	TBI, FSL-1	202.4 ± 28	89.7 ± 31.6	119.6 ± 14.7	262.5 ± 35.4
Ccl2/MCP-1 (1.8)	NT	ND	ND	ND	8.3 ± 6.5
	FSL-1	33.6 ± 8.4	12.2 ± 7.1‡	17.6 ± 1.8	8.3 ± 6.5
	TBI, NT	294.3 ± 291	6.4 ± 2.0	202.2 ± 193	ND
	TBI, FSL-1	170.3 ± 41	39.9 ± 3.3	46.8 ± 7.4	24.4 ± 6.6
Ccl3/MIP-1α (8.6)	NT	ND	ND	ND	22.3 ± 7.3
	FSL-1	12.8 ± 4.2	9.6 ± 1.0	23.4 ± 14.8	8.6
	TBI, NT	26.4 ± 3.8	10.3 ± 1.7	29.4 ± 20.8	ND
	TBI, FSL-1	64.4 ± 34.3	14.6 ± 2.6	8.6	10.3 ± 1.7
Ccl4/MIP-1β (8.2)	NT	ND	ND	ND	12.8 ± 4.6
	FSL-1	14.0 ± 2.9	11.8 ± 3.6	15.5 ± 3.9	9.0 ± 0.8
	TBI, NT	136.4 ± 115	12.0 ± 2.4	26.1 ± 14.5	ND
	TBI, FSL-1	34.7 ± 13.3	17.1 ± 4.2	18.4 ± 2.8	26.2 ± 4.6

Factors were measured by multiplex analyte assay MCYTOMAG-70K (EMD Millipore, Germany).

NT, no treatment; **p*<0.001, †*p*<0.01 and ‡*p*<0.05 for FSL-1 vs TBI, FSL-1.

ND, none detected.

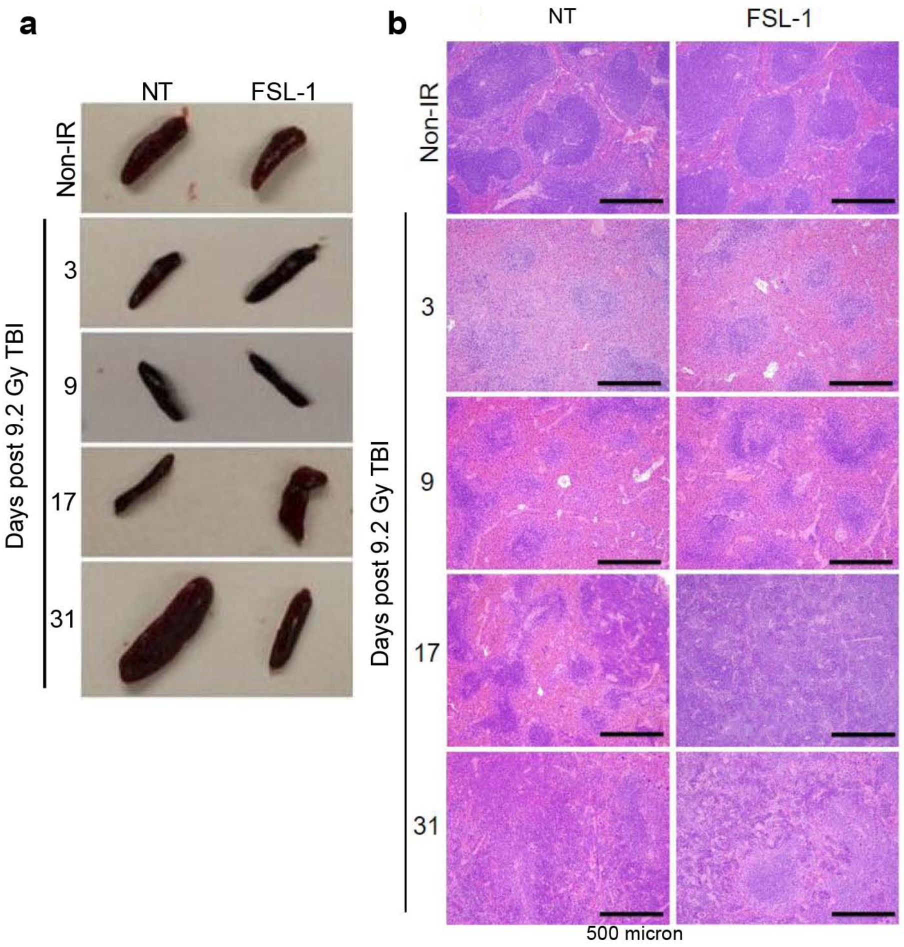

Suppl. Fig. S1. Splenic histology of FSL-1-treated mice. C57BL/6 mice were administered physiological water (NT) or FSL-1 at 24 hours post 9.2 Gy TBI (n=6-15 per group). Spleens were harvested at 3, 9, 17 and 31 days post TBI. (a) Representative pictures of spleens were taken at each time point. (b) Representative images of H&E stained spleen sections are shown.

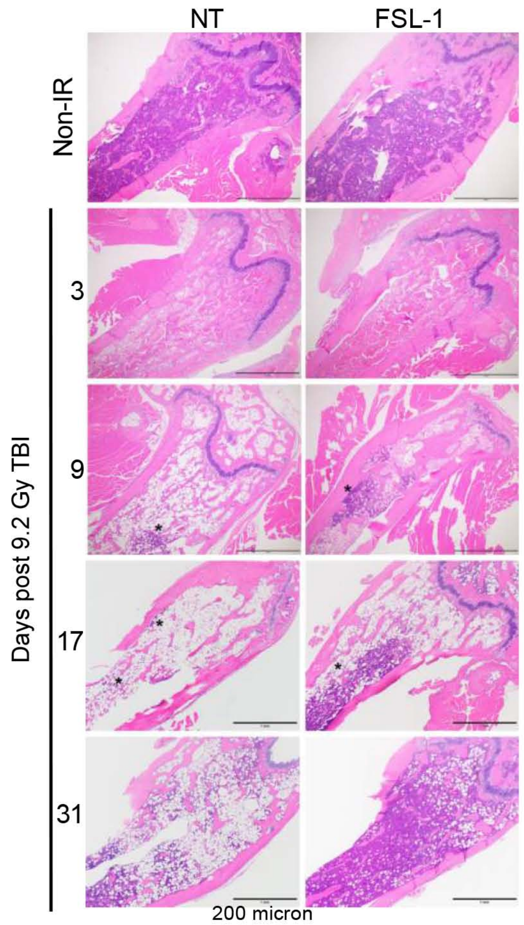

Suppl. Fig. S2. Femur histology of FSL-1-treated mice. Representative images of H&E stained femur sections are shown. * indicates areas of hematopoiesis.

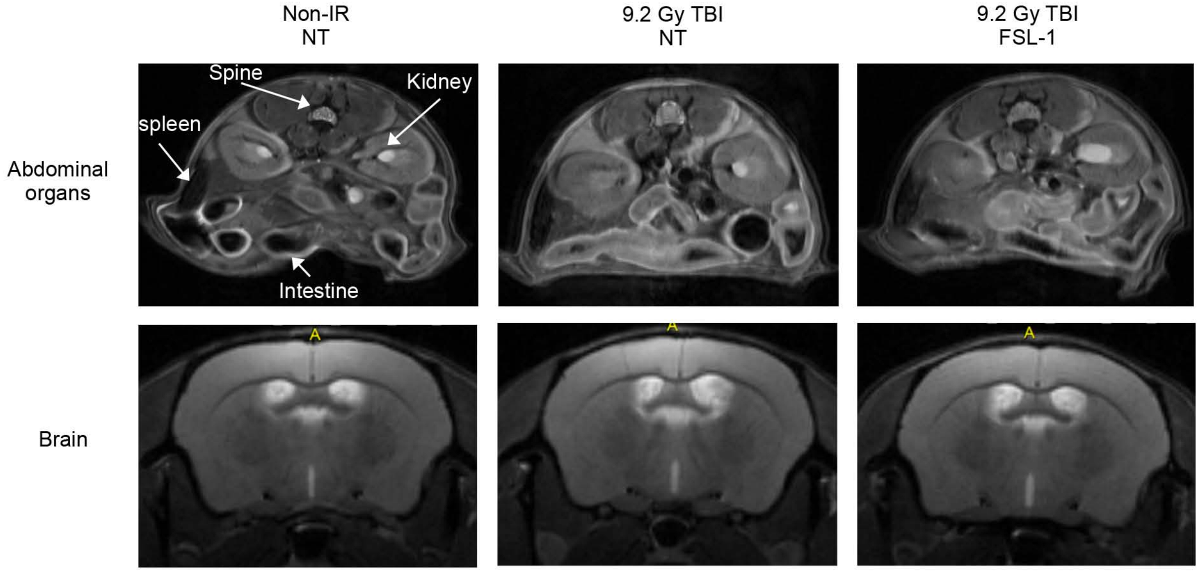

Suppl. Fig. S3. No physiologic changes evident in FSL-1-treated long-living survivors. Male C57BL/6 mice were administered physiological water (NT; 6/20 survived) or FSL-1 (19/20 survived) at 24 hours post 9.2 Gy TBI. Surviving mice were monitored for over 600 days and T2-weighted Magnetic Resonance Imaging (MRI) were conducted to three FSL-1 treated survivors and three age-matched control mice. A, axial view. Representative images of abdominal organs and brains are shown.
